# Supplementary material for: HPF1 remodels the active site of PARP1 to enable the serine ADP-ribosylation of histones
Source: Nat Commun. 2021 Feb 15;12:1028. doi: 10.1038/s41467-021-21302-4 (PMC7884425; doi:10.1038/s41467-021-21302-4)
Supplement: Supplementary file 1 — Supplementary Information [file 41467_2021_21302_MOESM1_ESM.pdf]

Supplementary Information

HPF1 remodels the active site of PARP1 to enable the serine ADP-ribosylation of histones

Fa-Hui Sun, Peng Zhao, Nan Zhang, Lu-Lu Kong, Catherine C L Wong and Cai-Hong Yun

Supplementary Tables

Table 1. Data collection and refinement statistics of crystal structures obtained in this study§

	H. sapiens HPF1 (Hg derivative)	*H. sapiens* HPF1 (Native)	*M. musculus* HPF1	*H. sapiens* HPF1/PARP1-CAT ΔHD
**PDB ID**		6M3G	6M3H	6M3I
**Data collection**				
Space group	*R32*	*R32*	$P2_12_12_1$	$P2_1$
Cell dimensions				
a, *b*, *c* (Å)	90.0, 90.0, 230.0	90.0, 90.0, 226.9	42.0, 79.9, 106.7	49.9, 84.2, 81.7
α, β, γ (°)	90.0, 90.0, 120.0	90.0, 90.0, 120.0	90.0, 90.0, 90.0	90.0, 107.5, 90.0
Resolution (Å)	50.0-2.36 (2.44-2.36)	50.0-1.57 (1.60-1.57)	50.0-1.71 (1.77-1.71)	50.0-1.98 (2.03-1.98)
R_{pim}*	0.035 (0.449)	0.017 (0.340)	0.023 (0.351)	0.053 (0.385)
CC1/2	0.997 (0.753)	0.998 (0.728)	1.002 (0.753)	0.989 (0.686)
I/σ	21.3 (2.0)	45.2 (2.2)	31.1 (2.1)	14.1 (2.1)
Completeness (%)	100.0 (100.0)	99.3 (85.1)	99.3 (94.9)	98.3 (99.0)
Redundancy	19.4 (16.7)	9.6 (8.4)	5.7 (4.1)	3.4 (3.4)
**Refinement**				
Resolution (Å)		45.9-1.57	44.4-1.71	27.4-1.98
No. reflections		49462	39364	44217
R_{work} / R_{free}		0.188/0.205	0.182/0.218	0.175/0.206
No. atoms				
Protein		2474	2493	4370
Ligand/ion		0	0	9
Water		341	274	323
B-factors (Å²)				
Protein		26.6	24.4	30.8
Ligand/ion		n/a	n/a	32.6
Water		34.3	33.5	34.8
R.m.s. deviations				
Bond lengths (Å)		0.008	0.007	0.008
Bond angles (°)		0.942	0.943	0.905
Ramachandran Plot				

Favored regions	96.97%	97.40%	97.97%
Allowed regions	3.03%	2.60%	2.03%
Outliers	0.00%	0.00%	0.00%

§Values in parentheses are for highest-resolution shell. One crystal was used for each data set.

*$R_{p.i.m.}$, a redundancy-independent R factor was used to evaluate the diffraction data quality as was proposed by Evans [1].

Supplementary Figures

Supplementary Figure 1. Architecture and overall structure of PARP1, as reported by Langelier *et al*. (PDB 4DQY)[2]. The overall structure of human PARP1 (Zn1, Zn3, WGR, HD and ART) binding to a DNA break is shown as cartoons. The automodification domain (AD) containing a BRCT domain was absent in this structure. Based on the remaining parts of PARP1 in the crystal structure, Langelier *et al*. proposed a location of AD/BRCT, which is shown in this figure.

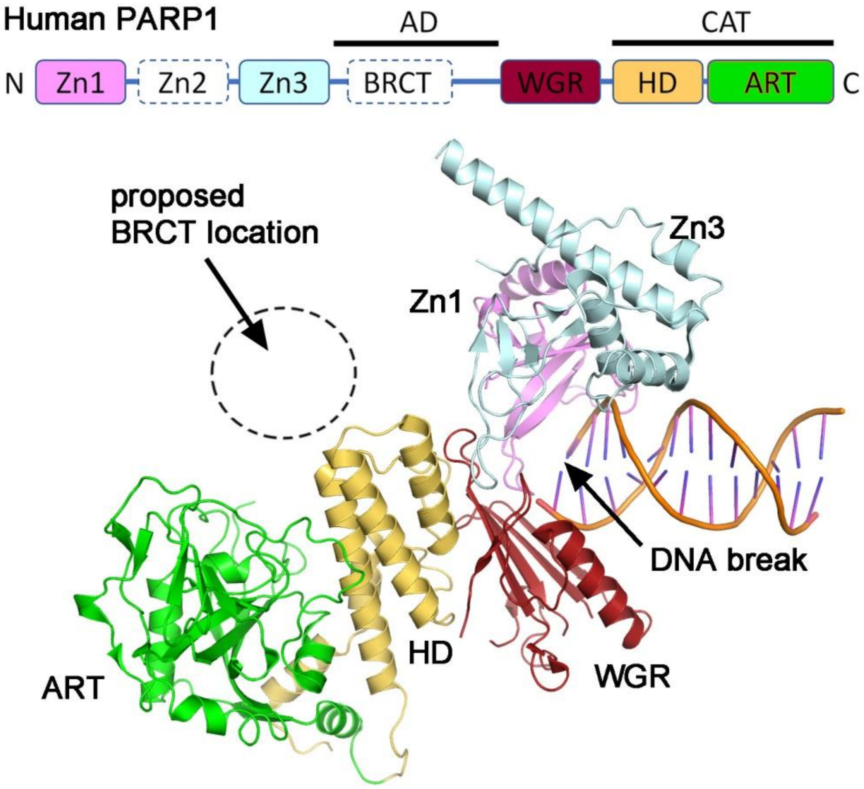

Supplementary Figure 2. Isothermal titration calorimetry (ITC) assays. The raw data of the ITC assays used to determine the K_d values shown in Table 1 are shown here. These assays were conducted in duplicate, except for full-length PARP1. Details of measurements are given in the "*Materials and Methods*" section.

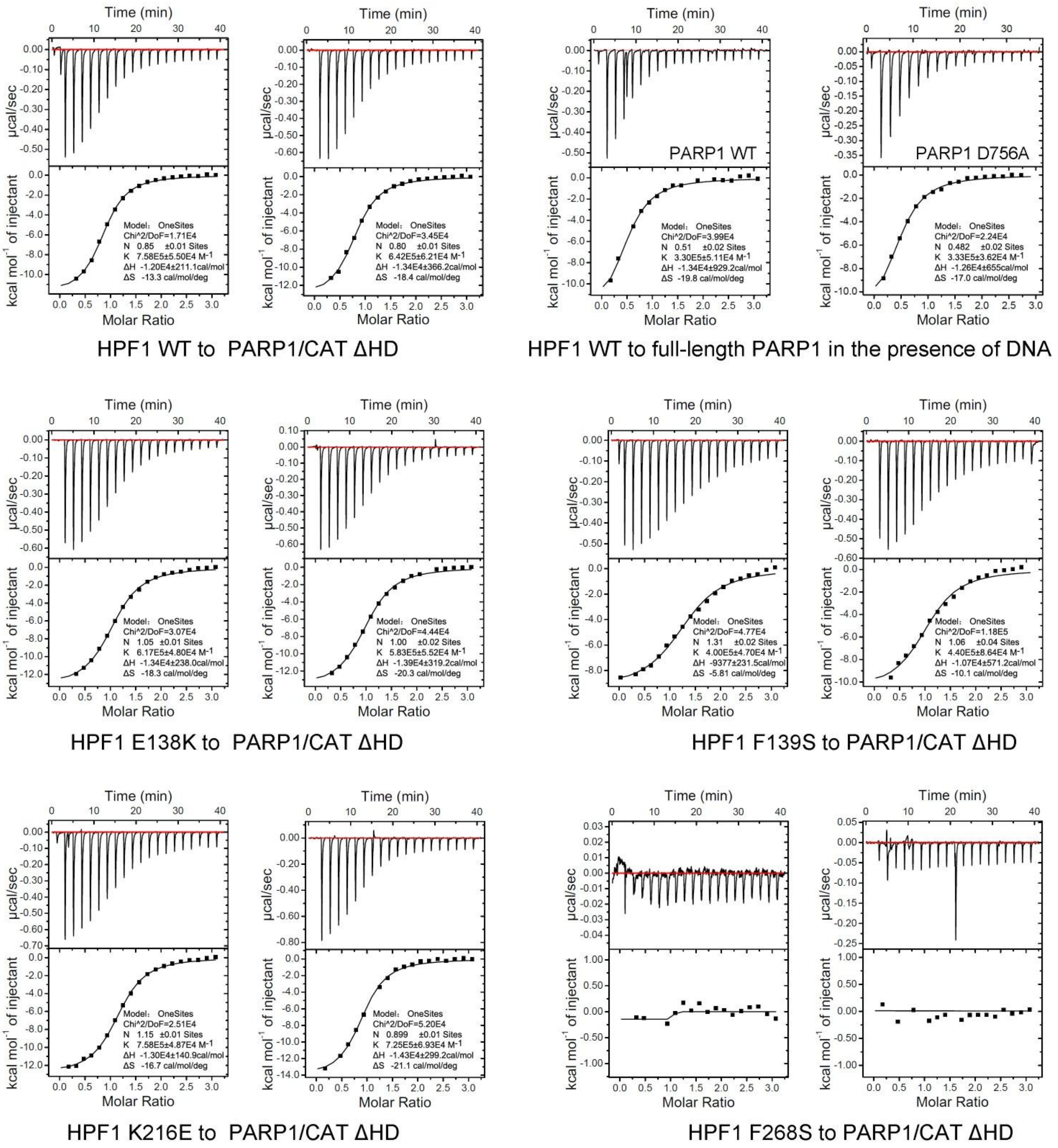

Supplementary Figure 3. Isothermal titration calorimetry (ITC) assays (continued). The raw data of the ITC assays used to determine the Kd values shown in Table 1 are shown here. These assays were conducted in duplicate. Details of measurements are given in the "*Materials and Methods*" section.

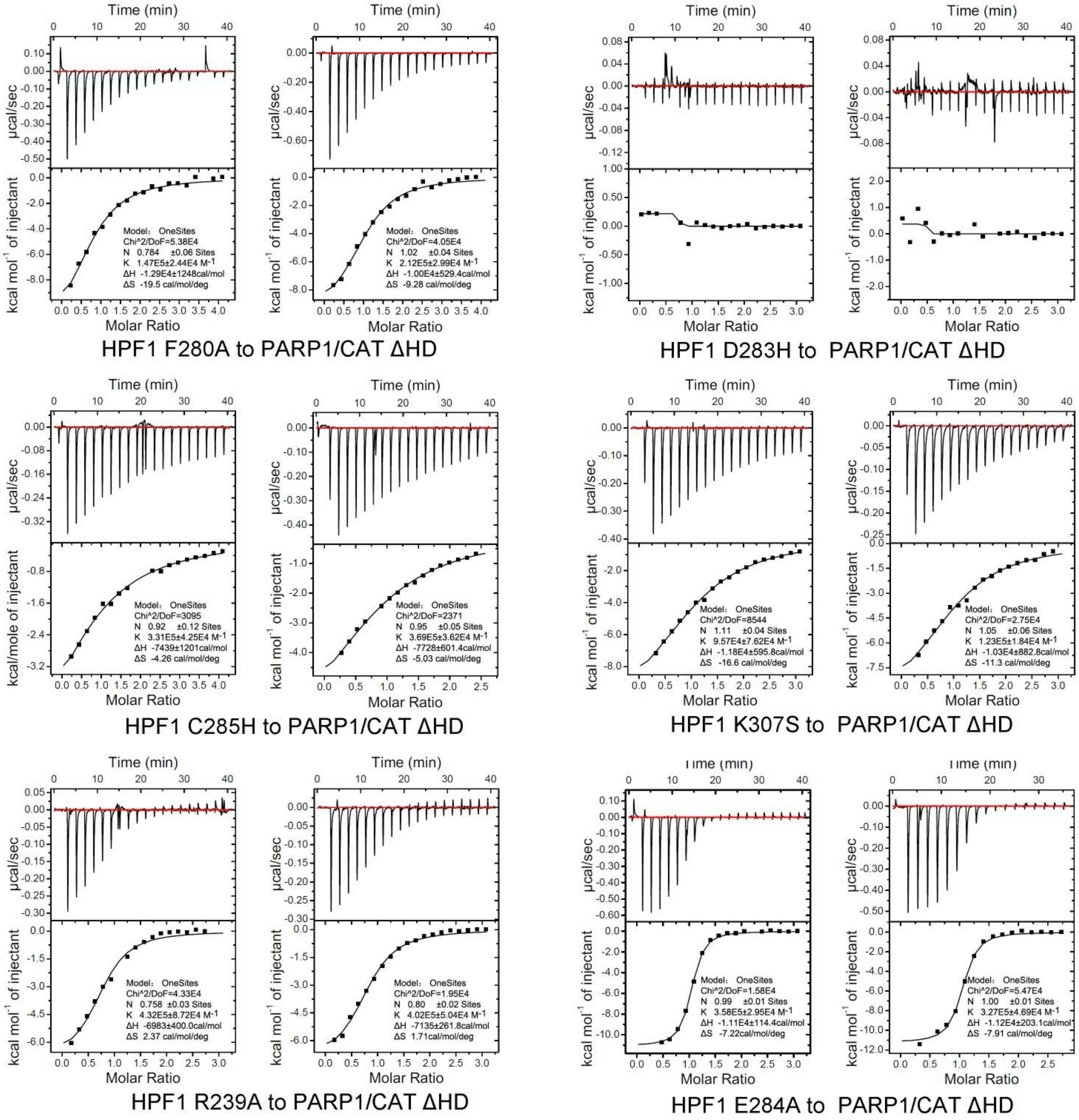

Supplementary Figure 4. Omit maps of the benzamide molecule in the active center of the HPF1/PARP1-CAT ΔHD crystal structure. The maps were calculated after removing the coordinates of benzamide and refining the structure by simulated annealing. Blue and green meshes showed the 2Fo-Fc and Fo-Fc electron density maps contoured at 1.0σ and +2.5σ level, respectively.

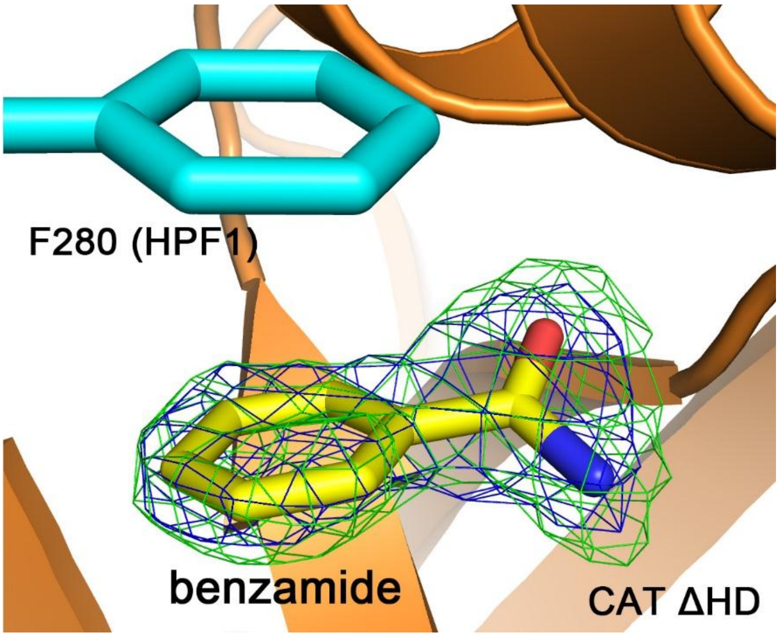

Supplementary Figure 5 Sequence alignment of HPF1 from representative species. Residues participating in inter-molecular interactions in interface I and II (see Figure 2a in main text) are annotated by round dots and triangles, respectively. Squares indicate the acidic amino acid residues contributing to the acidic/negatively charged surface covering helix α9, α10, and the loops connecting helices α6 and α7. Stars indicate Glu 284 and Arg 239.

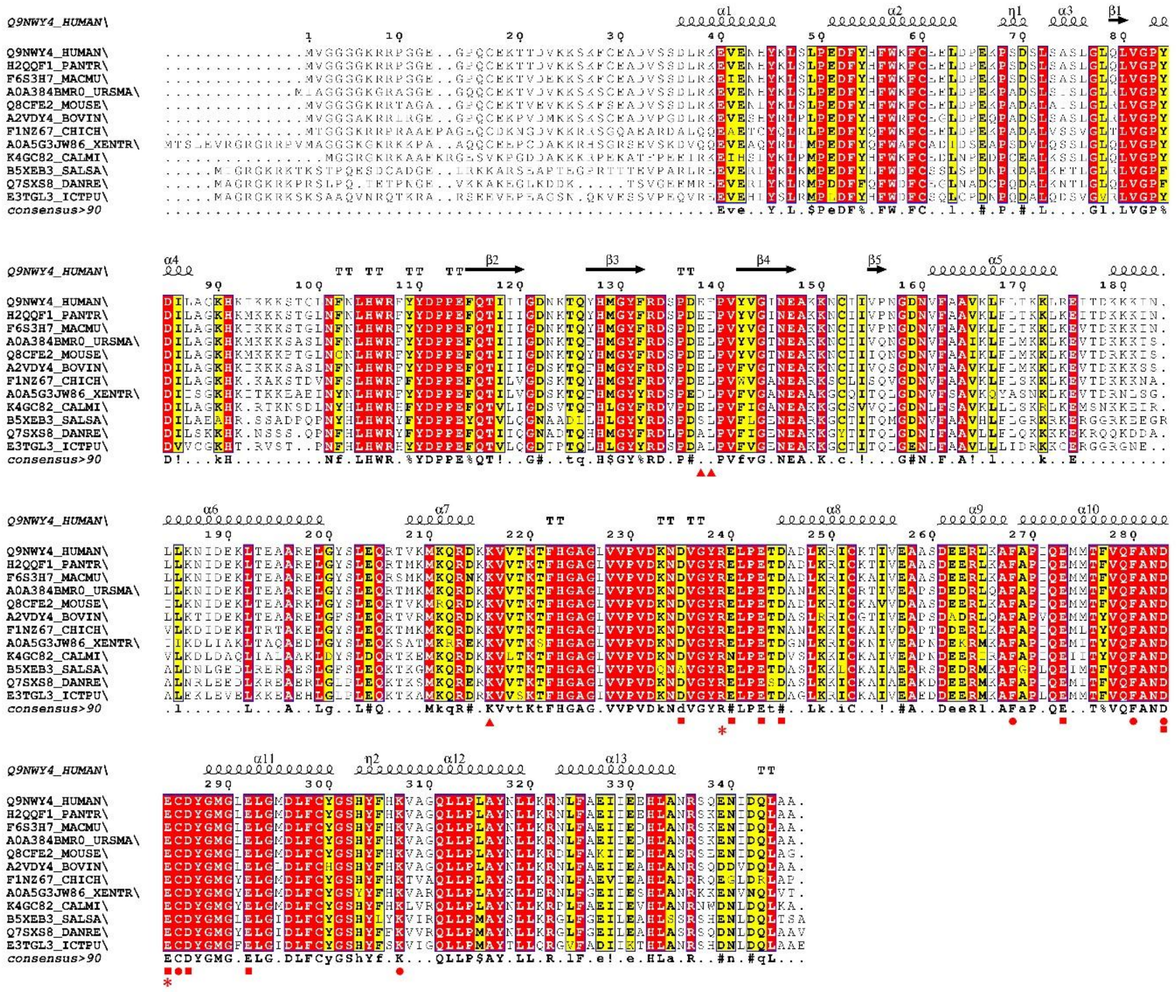

Supplementary Figure 6. Sequence alignment of human PARP1/2/3 CAT domain.

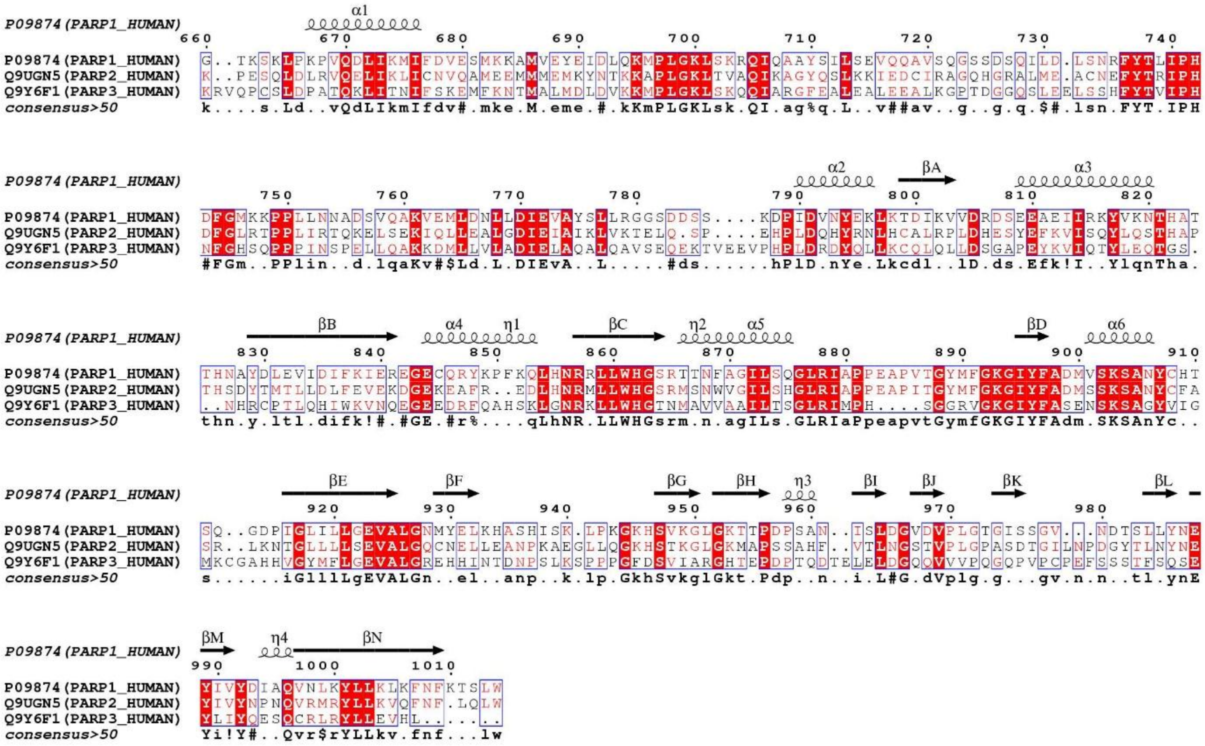

Supplementary Figure 7. Overall structure of HPF1. (a) Superimposition of human HPF1 (yellow) and mouse HPF1 (green) crystal structures. The protein structures are shown as cartoons in different colors. (b) Surface electrostatic potential of mouse HPF1. The surface potential was calculated using the PyMol APBS tools (http://pymolwiki.org/index.php/APBS). The positively and negatively charged areas are shown in blue and red, respectively.

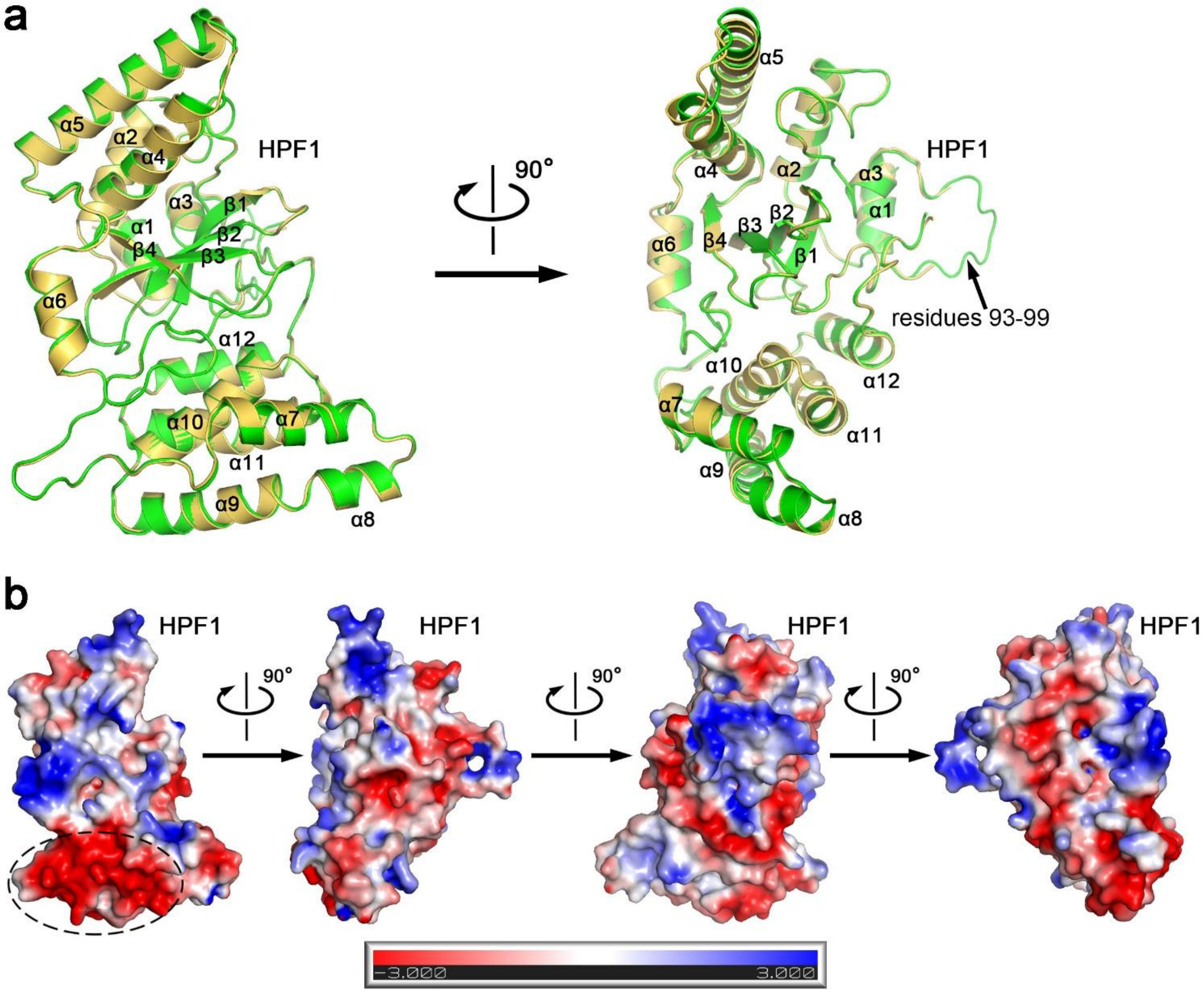

Supplementary Figure 8. Surface electrostatic potential of a nucleosome. The cartoon presentation of the same view is shown on the right-hand side for clarity. Histone/nucleosome ADP-ribosylation has been shown to preferentially take place at the Lys-Ser motif mainly residing on the N-terminal loop regions of histones[3, 4]. However, since the terminal loop regions of histones stretch out from the nucleosome and are flexible, to date no structure of human nucleosome showing these regions is available. We therefore constructed this figure using the *Xenopus laevis* nucleosome crystal structure (PDB 1KX5)[4], which is highly similar to that of human nucleosome. Most of the nucleosome/histone surface are basic/positively charged, including the putative ADP-ribosylation sites (the Lys-Ser motif, see labels and red arrows) that dominantly reside on H3 N-terminus, but also on H2A C-terminus and H2B N-terminus. The DNA double chains wrapping the nucleosome are shown as cartoons.

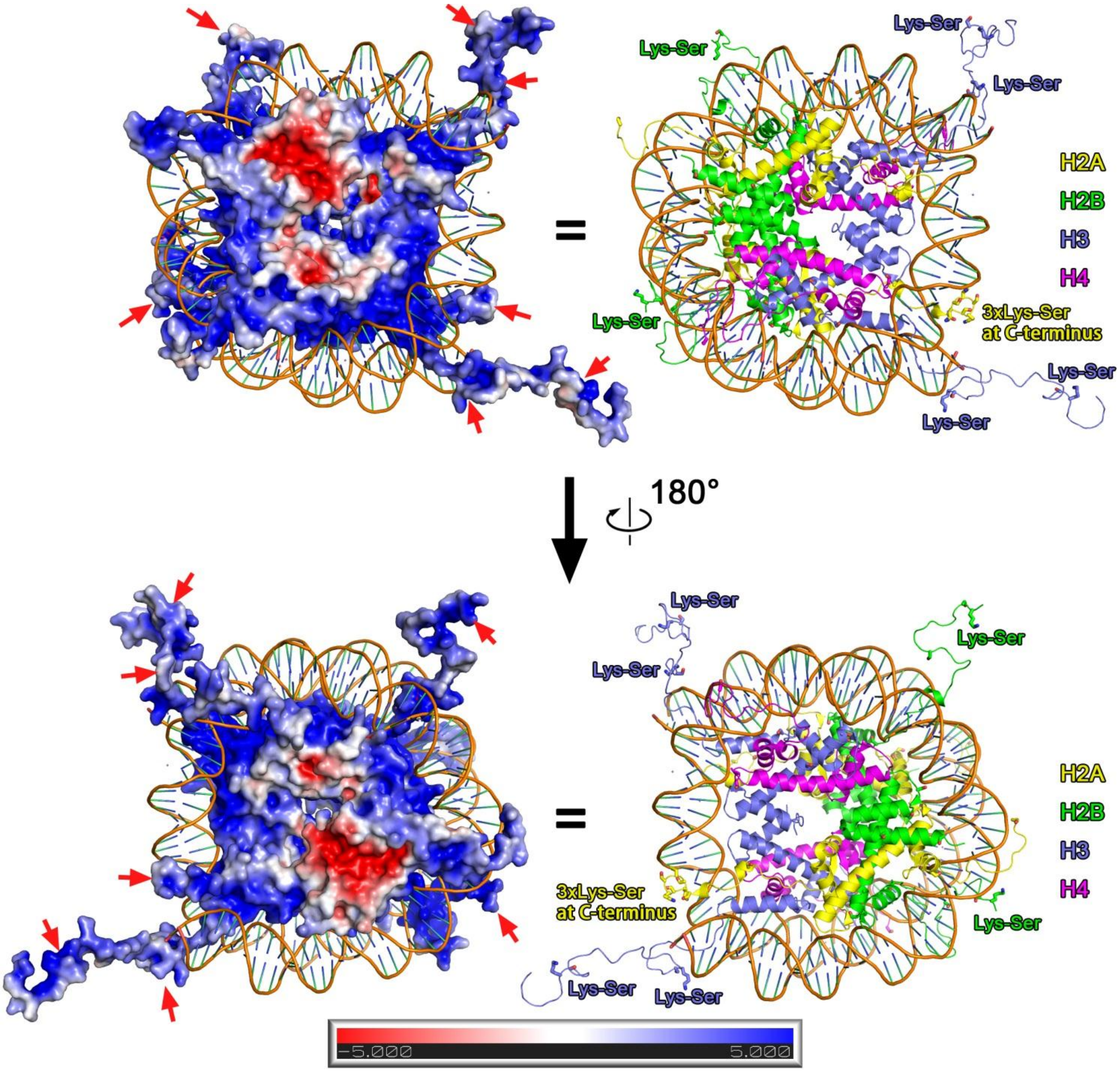

Supplementary Figure 9. Comparison of the HPF1 and HPF1/PARP-CAT ΔHD complex crystal structures determined by Suskiewicz *et al*.[5] and by us. The protein structures are shown as cartoons in different colors.

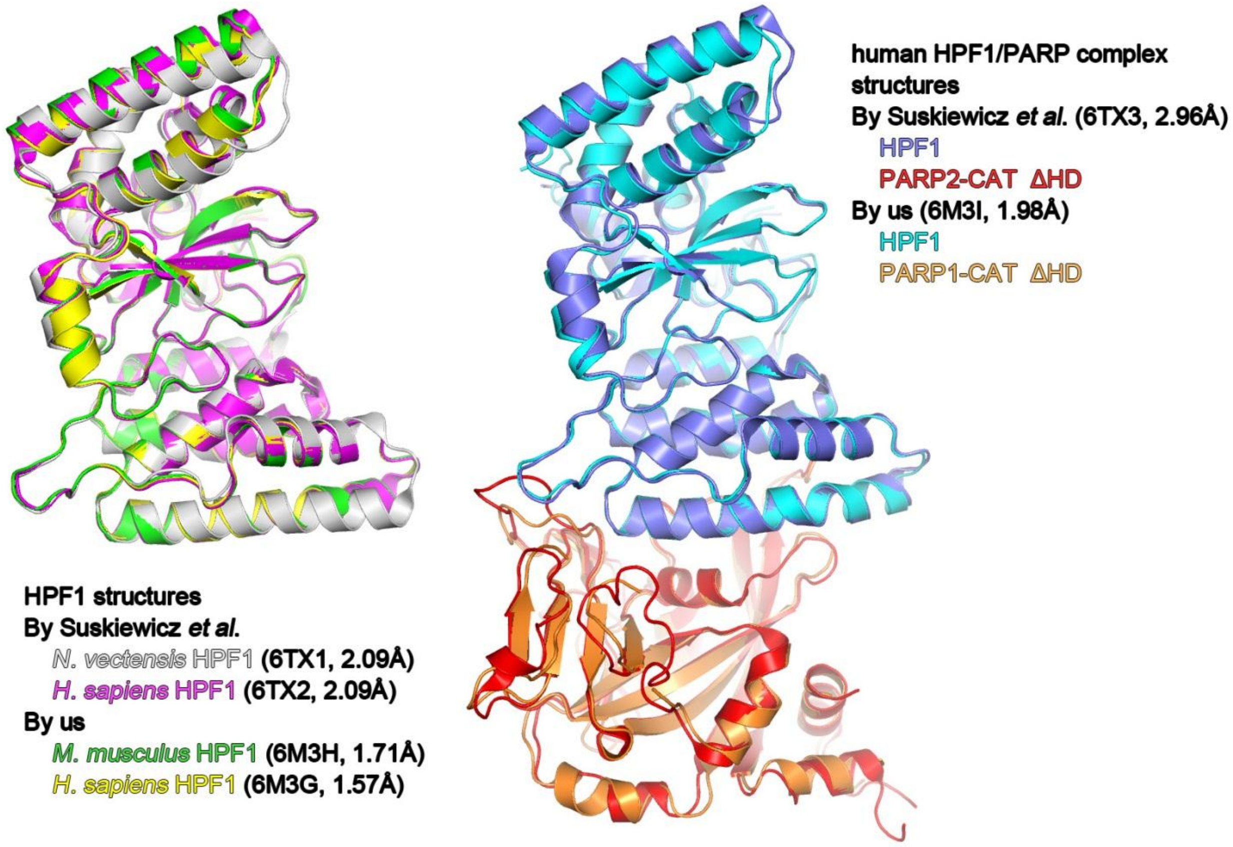

Supplementary References

1. Evans P. Scaling and assessment of data quality. *Acta Crystallogr D Biol Crystallogr* **62**, 72-82 (2006).

2. Langelier MF, Planck JL, Roy S, Pascal JM. Structural basis for DNA damage-dependent poly(ADP-ribosyl)ation by human PARP-1. *Science* **336**, 728-732 (2012).

3. Bonfiglio JJ*, et al.* Serine ADP-Ribosylation Depends on HPF1. *Mol Cell* **65**, 932-940 e936 (2017).

4. Palazzo L, Leidecker O, Prokhorova E, Dauben H, Matic I, Ahel I. Serine is the major residue for ADP-ribosylation upon DNA damage. *Elife* **7**, (2018).

5. Suskiewicz MJ*, et al.* HPF1 completes the PARP active site for DNA damage-induced ADP-ribosylation. *Nature* **579**, 598-602 (2020).
